# Relationship between MASC scores and diagnosis in a sample of children and adolescents in Spain

**DOI:** 10.1192/j.eurpsy.2024.958

**Published:** 2024-08-27

**Authors:** C. Canga-Espina, C. Vidal-Androher, A. Diez-Suarez, C. Maestro-Martin, M. Vallejo-Valdivielso

**Affiliations:** ^1^Child and Adolescent Psychiatry; ^2^Clinical Psychology, Clínica Universidad de Navarra, Pamplona, Spain

## Abstract

**Introduction:**

Anxiety is one of the most common Mental Health diagnosis in underage population. We decided to study if there was any variable that would lead us to a specific diagnosis, using the MASC questionnaire (*Multidimensional Anxiety Scale for Children*).

**Objectives:**

**1.** Describe the prevalence of the different anxiety disorders and the differences in its prevalence according to sex. **2.** Examine possible differences and associtions between MASC questionnaire scores and a specific anxiety diagnosis.

**Methods:**

This is a descriptive, observational, retrospective, quantitative study with data from patients between June 2016 and 2023. Inclusion criteria: 3-18 year-old-spanish-speakers who met criteria for a ICD-11 disorder. Exclusion criteria: absence of legal representatives, intellectual disability. **Variables:** sex, ICD-11 diagnosis, MASC’s subscales (Physical Symptoms, Harm Avoidance, Social Anxiety and Separation Anxiety) and CGI. **Statistical analyzes** were performed with STATA-15 program, using as independent variables MASC questionnaire and dependent one Anxiety Diagnosis.

**Results:**

The sample contains 1024 patients. Figure 1 shows the distribution of Anxiety Disorders: Unspecified Anxiety Disorder (47%), Separation Anxiety Disorder (23%), Simple Phobias (9%) and Social Anxiety Disorder (7%). Figure 2 represents the distribution by sex, with the differences being statistically significant (p<0.05) for all anxiety disorders, meaning that girls have higher prevalence of all anxiety disorders. Figure 3 shows how age correlates significantly and directly with all the subscales, meaning the older the patients are the higher the scores. We also found that boys have lower scores and a lower percentage of alteration in all subscales. CGI scale also correlates positively with all the subscales, specially with Physical Symptoms. All these data have been adjusted.

**Image:**

**Figure:**
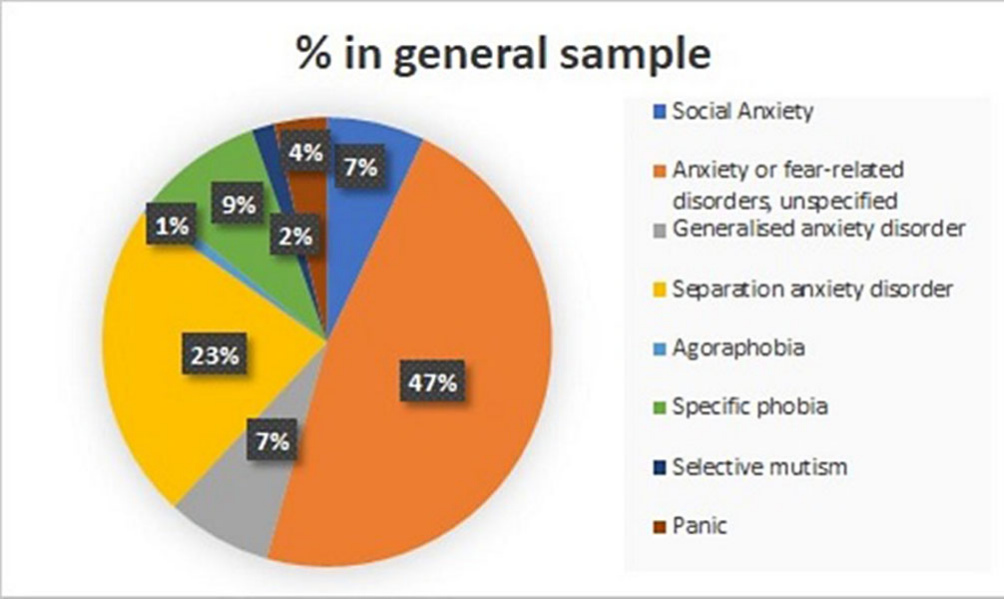

**Image 2:**

**Figure:**
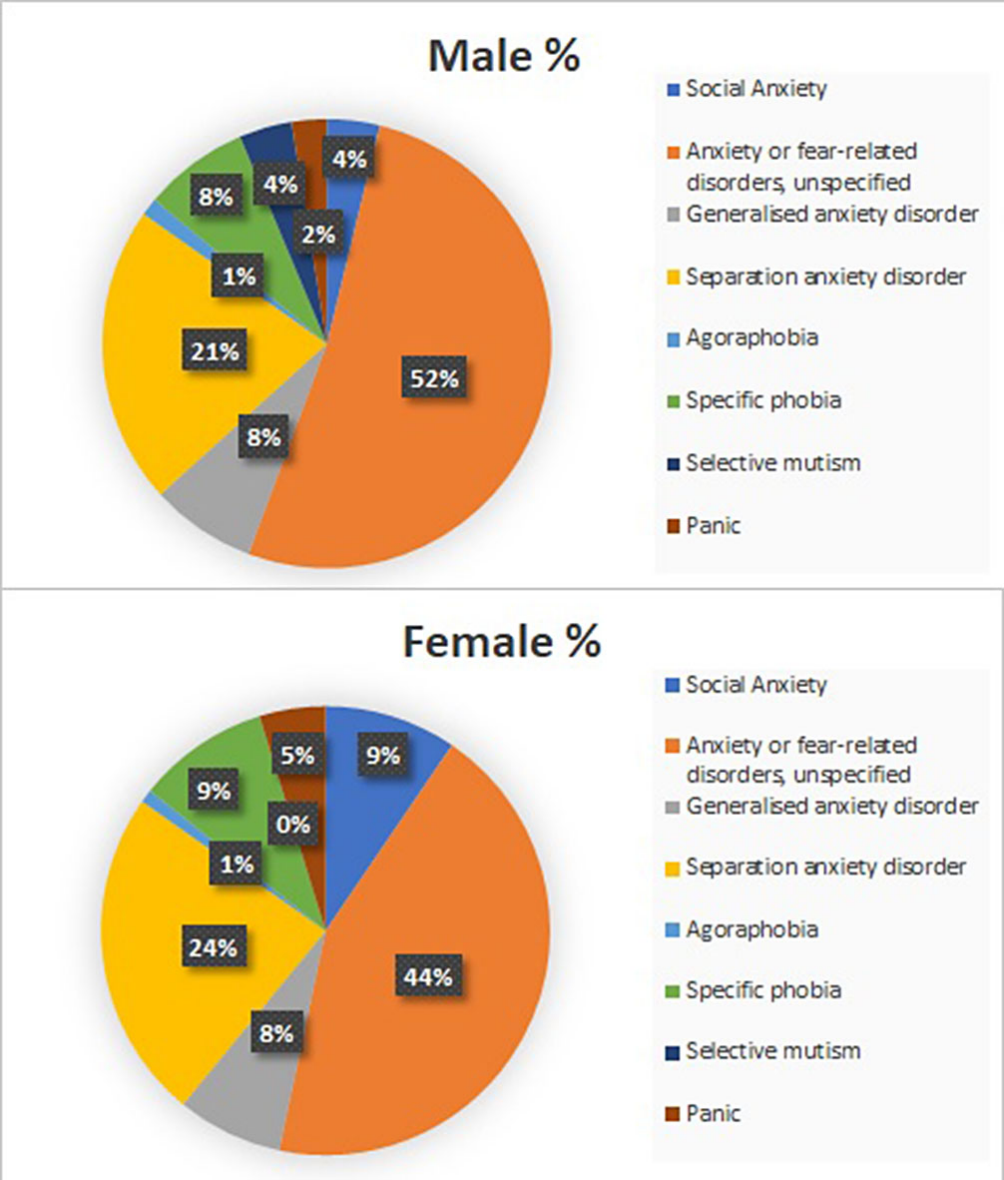

**Image 3:**

**Figure:**
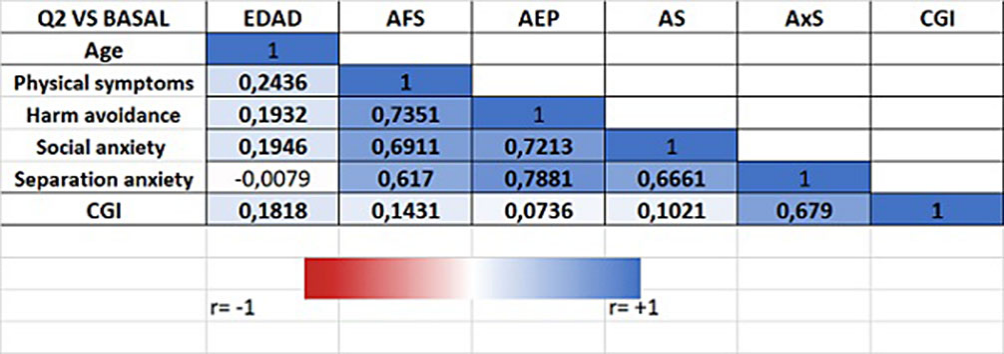

**Conclusions:**

Anxiety disorders are the most common form of Mental Disorder in young people (lobal prevalence of 6.5%, Rapee et al.2023). Prevalence for specific Anxiety Disorders in underage population are less reliable, because of the unequal age of samples (Rapee et al.2023). Separation Anxiety disorder is the most prevalent among children (La Maison et al., 2018), while Social Anxiety disorder is among adolescents (Lawrence et al.2015). We did not categorized our sample, being Separation Anxiety disorder the most frequent followed by Social Anxiety. We observed a correlation between some subscales and a specific diagnosis: the risk of presenting a Social Anxiety disorder is multiplied by 1.08 for each point of increase in that subscale and the risk of presenting a Separation Anxiety disorder is multiplied by 1.05 for each increase of 1 point in Separation Anxiety subscale. However, the diagnosis of Simple Phobia decreases with the increase in scores in all subscales, maybe due to the fact that there are not many items that specifically evaluate fears.

**Disclosure of Interest:**

None Declared

